# CYP1B1-catalyzed 4-OHE2 promotes the castration resistance of prostate cancer stem cells by estrogen receptor α-mediated IL6 activation

**DOI:** 10.1186/s12964-021-00807-x

**Published:** 2022-03-15

**Authors:** Qimei Lin, Jiasong Cao, Xiaoling Du, Kuo Yang, Xu Yang, Zhixian Liang, Jiandang Shi, Ju Zhang

**Affiliations:** 1 Department of Biochemistry and Molecular Biology, College of Life Sciences, Bioactive Materials Key Lab of the Ministry of Education, Nankai University, Tianjin, 300071 China; 2 Tianjin Key Lab of Human Development and Reproductive Regulation, Tianjin Central Hospital of Obstetrics and Gynecology, Nankai University, Tianjin, 300071 China; 3 Department of Urology, Second Hospital of Tianjin Medical University, Tianjin, 300211 China

**Keywords:** CYP1B1, 4-OHE2, ERα, IL6-STAT3 pathway, PCSC, Castration-resistant prostate cancer

## Abstract

**Background:**

Resistance to androgen deprivation therapy remains a major challenge for the clinical treatment of patients with castration-resistant prostate cancer (CRPC). CYP1B1, a critical enzyme that catalyzes the conversion of estradiol to 4-Hydroxy-17β-estradiol (4-OHE2), has been reported to promote the development and progression of hormone-related cancer, but its role in CRPC is unclear.

**Methods:**

To explore the underlying mechanism which CYP1B1 promotes the prostate cancer stem cells (PCSCs) characteristics, bioinformatics analyses of human clinical prostate cancer (PCa) datasets were performed. CYP1B1, IL6, and estrogen receptor-α (ERα) expression levels were evaluated in PCa and CRPC tissues via immunohistochemistry. The high-performance liquid chromatography-mass spectrometry assay was carried out to examine intracellular 4-OHE2 levels. Serum-free suspension culture and flow cytometry assays were performed to evaluate PCSCs. Chromatin immunoprecipitation was used to validate that 4-OHE2 recruited ERα to the IL6 promoter.

**Results:**

CYP1B1 expression was significantly increased in CRPC tissues and androgen-independent PCa cell lines. CYP1B1^+^ PCa cells were significantly enriched in bicalutamide-treated LNCaP cells, and CYP1B1 knockdown reduced the cell viability under bicalutamide treatment. In addition, CYP1B1 knockdown decreased the intracellular 4-OHE2 concentration, accompanied by reduced PCSC characteristics. In PCa cells, 4-OHE2 stimulated ERα transcriptional activity and upregulated the expression of IL6 and downstream genes of the IL6-STAT3 signaling. 4-OHE2 increased cell viability under bicalutamide treatment and promoted PCSC characteristics, while IL6 neutralizing antibody reversed these effects. Mechanistically, siERα and the ER antagonist ICI182780 significantly attenuated 4-OHE2-induced IL6 expression, and 4-OHE2 promoted the binding of ERα to the estrogen response element of the *IL6* promoter.

**Conclusions:**

Our findings indicate that CYP1B1-catalyzed 4-OHE2 enhanced PCSC characteristics and attenuated bicalutamide sensitivity by ERα-mediated the IL6-STAT3 pathway activation. Our study further emphasizes the role of CYP1B1 in castration resistance and illustrates a novel mechanism of CRPC development.

**Graphical Abstract:**

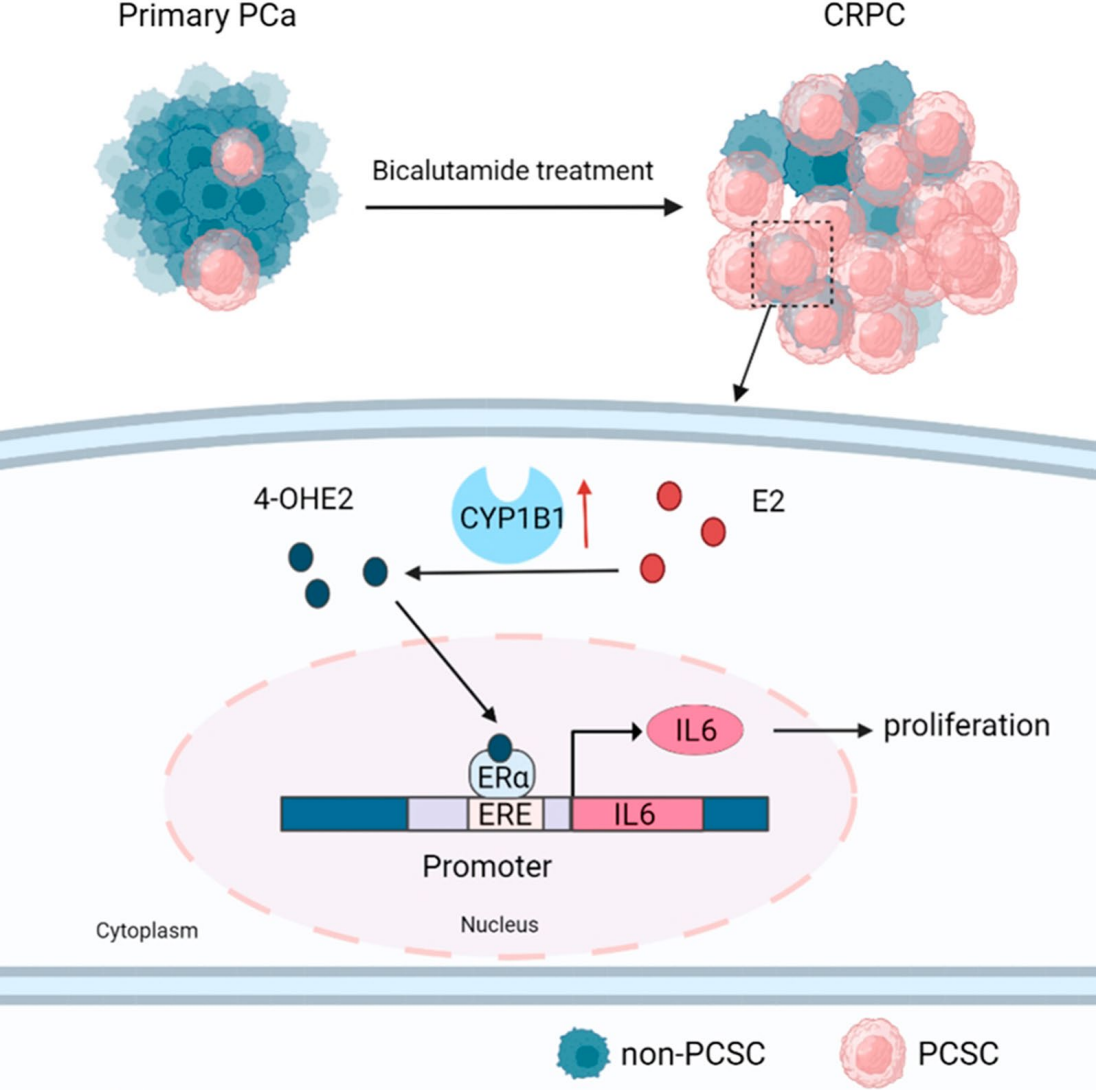

**Video Abstract**.

**Supplementary Information:**

The online version contains supplementary material available at 10.1186/s12964-021-00807-x.

## Background

Prostate cancer (PCa) is the most common cancer and the second leading cause of cancer-related death in men [1]. The growth of PCa cells relies on androgens through binding to the androgen receptor (AR) [2, 3]. Therefore, androgen deprivation therapy (ADT), including surgical or chemical castration that is induced by nonsteroidal androgen receptor antagonists such as bicalutamide, is currently the standard clinical treatment for advanced prostate cancer [4, 5]. Although the growth of PCa can be inhibited in the short term with bicalutamide treatment, it still inevitably progresses to castration-resistant prostate cancer (CRPC) [6]. This process may be related to the abnormal activation of the androgen receptor in CRPC and the restoration of part of the androgen receptor signaling pathway mediated by the appearance of AR mutants [7, 8]. Indeed, sustained androgen receptor signal activation plays an important role in promoting the progression of primary PCa, but treatments targeting AR signals seem to be insufficient to significantly improve the survival rate of CRPC patients [9, 10]. Therefore, it is very important to explore the resistance mechanism of CRPC from a new perspective.

Increasing efforts suggest that estrogen and its receptor downstream signaling pathways are implicated in CRPC development [11–13]. PCa patients with higher estrogen receptor-α (ERα) expression display poorer distant metastasis-free survival after ADT treatment [14]. Estradiol enhances the PCSC phenotype through cross-talk with the Notch1, hypoxia, and β-catenin signaling pathways [15–17]. In contrast, tamoxifen, an estrogen receptor antagonist, could induce the apoptosis of PC3 cells and suppress tumor growth in PCa xenograft models [18]. On the other hand, it has been reported that 4-hydroxyestradiol (4-OHE2), a metabolite of estradiol, plays a critical role in the occurrence and progression of hormone-related cancers [19]. 4-OHE2 is highly carcinogenic in a variety of malignant tumors, including endometrial cancer [20] and breast cancer [21, 22]. 4-OHE2 induces anchorage-independent growth of human mammary epithelial cells [23]. In prostate epithelial cells, 4-OHE2 is more carcinogenic than the parent hormone estradiol [24].

CYP1B1 (cytochrome P450, family 1, subfamily B, polypeptide 1) is an enzyme that catalyzes 17β-estradiol to 4-OHE2 [25]. CYP1B1 is abnormally highly expressed in a variety of cancers (e.g., breast cancer, head and neck cancer, and PCa), and its effect on reducing the drug sensitivity of tumor cells has been widely reported [26–28]. For example, in gastric cancer, overexpression of CYP1B1 can increase its resistance to cisplatin [29]. Meanwhile, elevated CYP1B1 levels enhance the resistance of ovarian and prostate cancer cells to paclitaxel [30, 31]. In particular, the role and mechanism of CYP1B1 in the treatment failure of bicalutamide and in the progression of CRPC have not been reported.

Prostate cancer stem cells (PCSCs) play a critical role in CRPC progression [32, 33]. It has been reported that the activation of the estrogen signaling pathway is associated with the enrichment of PCSCs [34]. Our previous studies have shown that estradiol promotes the PCSC phenotype by activating the Notch1 signaling pathway [17]. However, little is known about the role of 4-OHE2 in promoting PCSC characteristics.

In this study, we explored the role of the estradiol metabolite 4-OHE2 in CRPC. We identified that increased CYP1B1 expression in PCa cells could elevate endogenous 4-OHE2 concentration and enhance CD44^+^/CD24^−^ PCSC characteristics, thereby promoting castration resistance. Correspondingly, 4-OHE2 could also attenuate the sensitivity of PCa cells to bicalutamide. Mechanistically, 4-OHE2 promoted IL6 expression via ERα in androgen-independent PCa cells. Ablation of IL6 antagonized 4-OHE2-induced PCSC characteristics and reversed the 4-OHE2-induced decrease in bicalutamide resistance.

## Methods

### Cell culture and treatment

The PCa cell lines PC3 and Du145 were obtained from the Deutsche Sammlung fuer Mikroorganismen and Zellkulture (DSMZ, Braunschweig, Germany). LNCaP and LNCaP-abl cells were gifts from Professor Helmut Klocker (the Innsbruck University School of Medicine). LNCaP and PC3 cells were maintained and propagated in RPMI-1640 medium (Sigma-Aldrich, Saint Louis, Missouri, USA) supplemented with 10% fetal bovine serum (FBS; Invitrogen, Carlsbad, CA, USA) and 1% 100 mg/ml penicillin/streptomycin (P/S, HyClone, Logan, UT, USA). LNCaP-abl cells were cultured in RPMI-1640 medium supplemented with 100 mg/ml P/S and 10% charcoal dextran stripped fetal bovine serum (CS-FBS; Invitrogen). Cells were routinely cultured in a 5% CO_2_, 37 °C incubator. 4-Hydroxy-17β-estradiol (4-OHE2; Toronto Research Chemicals, Canada) was used at a concentration of 10 nM. Bicalutamide (Selleck Chemicals, Houston, Texas, USA) was used at 20 μM. IL6 neutralizing antibody (Sino Biological, Beijing, China) was used at 100 ng/mL. Recombinant IL6 protein (Proteintech, Chicago, USA) was used at 10 ng/ml.

### Quantitative RT-PCR and western blot analysis

According to the manufacturer’s instruction, total RNA and protein from cells were extracted and collected using TRIzol reagent (Invitrogen) and RIPA lysis buffer (Thermo Fisher, Waltham, MA, USA), respectively. qRT-PCR and western blotting procedures were performed as described previously [35]. The primer sequences used here are listed in Additional file 1: Table S1, and all antibodies used in western blotting are shown in Additional file 1: Table S2.

### Immunohistochemistry (IHC) and Immunofluorescence (IF) analysis

Human benign prostate tissues from patients (n = 5) undergoing radical cystectomy for bladder cancer were acquired from the Department of Urology, Shanghai First People’s Hospital (Shanghai, China). PCa tissues from patients (Gleason scores of 2–10, n = 20) undergoing radical prostatectomy and CRPC tissues from patients (n = 11) undergoing radical prostatectomy for prostate carcinoma were acquired from the Department of Urology of the Second Hospital, Tianjin Medical University (Tianjin, China). All patients provided written informed consent, and the present study was approved by the Ethics Committee of Nankai University.

IHC was carried out on the paraformaldehyde-fixed paraffin sections. The IHC procedure was performed according to a previous study [35]. The following primary antibodies were used: rabbit anti-CYP1B1 (1:500, Abcam, ab32649, UK), rabbit anti-IL6 (1:100, Abcam, ab6672), and rabbit anti-ERα (1:500, Abcam, ab32063). Histologic images were visualized under an Olympus BX43 microscope (Olympus, Tokyo, Japan) at 100 × and 400 × magnification, and the optical density of the image was analyzed using ImageJ software.

For IF assays, PCa cell lines were grown on a coverslip placed in a 24-well plate and treated as indicated. The IF procedure was performed as described previously [35]. Primary antibodies are displayed in Additional file 1: Table S2. The secondary antibodies used here were Alexa-488 and rhodamine and conjugated to specific IgG types that matched the primary antibodies. Nucleus were counterstained with DAPI. Immunofluorescent images were observed and photographed using a fluorescence microscope (Leica, Germany) with 20 × and 40 × objective.

### Cell growth and invasion assays

For cell growth assays, cells were treated with the indicated compound. The total cell numbers were counted, and the cell survival rate [(treatment group cell number/control group cell number) *100%] was calculated after 3 days.

Cell invasion assays were performed using Transwell chambers with filter membranes of 8-μm pore size (BD Falcon) coated with Matrigel (1:3 dilution; BD Biosciences; San Jose, CA, USA). Cells received the indicated treatment and were resuspended in 100 μl serum-free RPMI-1640 medium and seeded in Transwell inserts. Transwell inserts were placed in 24-well plates with 10% FBS 1640. After incubation for 24 h at 37 °C in a 5% CO_2_ incubator, cells on the lower surface of the filter membrane were stained with crystal violet (BBI, Shanghai, China) and counted under a light microscope.

### Plasmid construction

The *CYP1B1* full-length coding sequence was incorporated into pcDNA3.1( +) mammalian expression vectors to obtain the pcDNA3.1( +)-CYP1B1 construct. The recombinant pcDNA3.1( +)-CYP1B1 plasmid was confirmed by sequencing. The CRISPRi sgRNA sequence targeting the human *CYP1B1* gene was subcloned into the letiSAM2-dCas9-KRAB vector to obtain CRISPRi-CYP1B1. A control sgRNA oligonucleotide whose sequence did not match any known human DNA, was used as a control. The plasmid construction process and plasmid maps are shown in Additional file 2: Fig. S1. The cloning primers are shown in Additional file 1: Table S3.

### Transient and stable transfection

Cells were cultured in a 6-well plate. According to the manufacturer’s protocol and the result of plasmids transfection efficiency/cytotoxicity (Additional file 2: Fig. S2), plasmids were transfected into cells using Lipofectamine 3000 (Invitrogen, Carlsbad, CA, USA) when the cells reached 50–70% confluence. For lentivirus packaging, HEK293T cells were transfected with CRISPRi-CYP1B1 plasmids or control CRISPRi plasmids and viral packaging plasmids (psPAX-2 and pMD2.G) using Lipofectamine 3000 to generate CRISPRi-CYP1B1 virus or control CRISPRi virus. After 48 h, the supernatant was collected, and the lentiviral particles were harvested by centrifugation (3000 rpm, 10 min at 4 °C). Then, particles were filtered through a 0.22 µm filter, and the viral concentrate was collected by filtrate centrifugation for 90 min at 20,000 rpm at 4 °C. To generate PC3 and LNCaP-abl cell populations with stable CYP1B1 knockdown, lentivirus-mediated vectors were infected into PC3 or LNCaP-abl cells. After 72 h of infection, PC3 or LNCaP-abl cells were treated with blasticidin (Solarbio, Beijing, China) for 7 days to select stable cell lines. Effective knockdown was confirmed by qRT-PCR and western blot analysis.

### Dual-luciferase assays and high–performance liquid chromatography-mass spectrometry (HPLC–MS) assay

Cells were seeded into 24-well plates and then treated as indicated. PGL3-(ERE)6-luciferase reporter (ERE-Luc) and pGL3-IL6-promoter-luciferase reporter (IL6-pro-Luc) were transfected into cells. Then, the luciferase activity was measured by the dual-luciferase reporter gene detection kit (Promega) according to the manufacturer's protocol. For the HPLC–MS assay, 1*10^6 cells after the indicated treatment were suspended in 1 mL PBS supplemented with 2 μg/mL leupeptin, 0.5 mM PMSF, 0.5 mM DTT, 1 mM sodium orthovanadate, and 0.1% L-ascorbic acid and were used to extract and derive 4-OHE2 as described [36]. The intracellular 4-OHE2 concentration was measured by liquid chromatography-tandem mass spectrometry (LCMS-2020, SHIMADZU, Japan). The monitored ion of 4-OHE2 on the mass spectra was at 755 m/z, and the retention time was 6.95 min (Additional file 2: Fig. S3).

### Chromatin immunoprecipitation (ChIP) assay

PC3 and LNCaP-abl cells were seeded in 100-mm dishes and treated with DMSO or 10 nM 4-OHE2 for 30 min before harvest. ChIP assays were performed as described previously [17], and ERα antibody (Abcam, ab32063) was used. The following primers were used: forward: 5'- GAGTGGTTCTGCTTCTTAGCG -3'; reverse: 5'- TGAGCCTCAGACATCTCCAGT -3.

### Flow cytometry assay

PC3 and LNCaP-abl cells were washed in PBS and collected by centrifugation. Flow cytometry was carried out by standard procedures using the following fluorochrome-conjugated antibodies: FITC anti-human CD44 (1:20; BioLegend, San Diego, CA, USA) and PE anti-human CD24 (1:20; BioLegend). Fluorescence analysis was performed using a FACSCalibur flow cytometer (BD, San Jose, CA, USA).

### Tumorsphere formation assay

LNCaP-abl cells were digested, and 2000 cells were seeded into ultralow attachment 6-well plates (Corning Inc., Life Sciences, USA) with DMEM/F-12 (HyClone) serum-free medium containing 1 μL/mL transferrin (Sigma-Aldrich, USA), 20 ng/mL EGF (PeproTech, USA), 20 ng/mL basic FGF (PeproTech), 2% B27 (Invitrogen, USA), and 10 unit/mL human LIF (Sigma-Aldrich). The formed tumorspheres were counted by inverse microscopy after 2 weeks.

### Bioinformatic analysis

The Roudier dataset (GSE74367) [37] from the Gene Expression Omnibus (GEO, https://www.ncbi.nlm.nih.gov/geo/) and Chandran [38] and Tamura [39] datasets from the Oncomine (http://www.oncomine.org/) were used in our study. Correlation analysis was performed in GEPIA2 (http://gepia2.cancer-pku.cn/). RNA-seq data from 498 PCa cases were downloaded from The Cancer Genome Atlas (TCGA) database. The samples in the top and bottom 10% according to CYP1B1 expression levels were selected and normalized. Heatmaps and hierarchical clustering were acquired from Morpheus (http://software.broadinstitute.org/morpheus). Gene set enrichment analysis (GSEA) was carried out using GSEA 3.0.

### Statistical analysis

Each experiment was performed at least in triplicate, and all data are presented as the means ± standard deviation. Data analysis was performed by one-way analysis of variance (ANOVA) for multiple comparisons of groups. For comparisons between 2 groups, Student’s t-test was used. Differences with *p*-values < 0.05 were considered to be statistically significant (*p < 0.05, **p < 0.01, and ***p < 0.001).

## Results

### CYP1B1 is highly expressed and involved in the regulation of bicalutamide sensitivity in CRPC

**Fig. 1:**
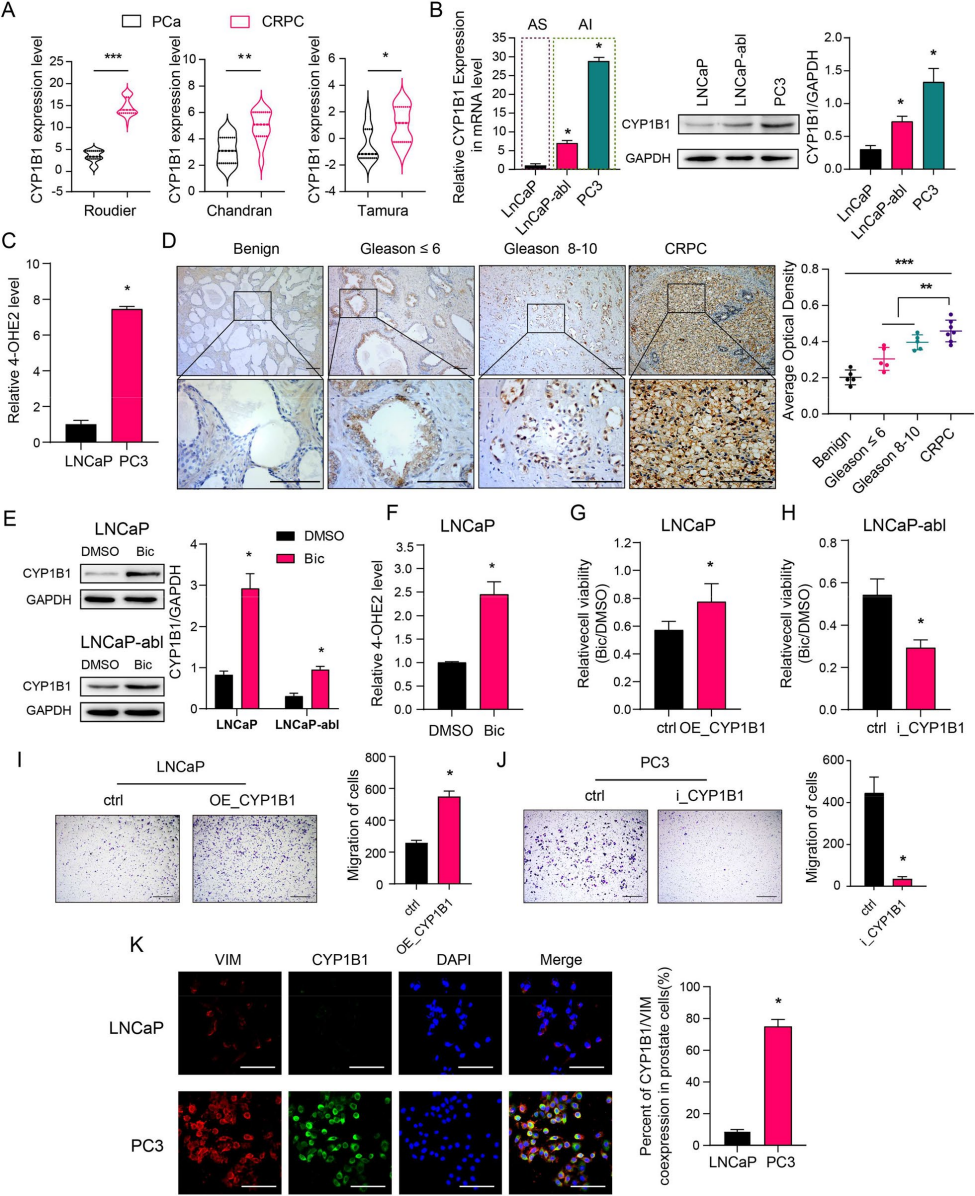
CYP1B1 is highly expressed and involved in the regulation of bicalutamide sensitivity in CRPC. **A** Scatter dot plots present *CYP1B1* mRNA expression in prostate tumor and CRPC tissues from 3 publicly available datasets (Roudier, n(PCa) = 11, n(CRPC) = 45; Chandran, n(PCa) = 10, n(CRPC) = 13; Tamura, n(PCa) = 8, n(CRPC) = 17) (t-test). **B** Basal mRNA and protein levels of CYP1B1 in different PCa cell lines (one-way ANOVA). **C** The concentration of intracellular 4-OHE2 in LNCaP and PC3 cells (t-test). **D** Left: Representative IHC staining of CYP1B1 protein in benign prostate tissues, primary PCa and CRPC tissues. Right: Quantification of the average optical density for CYP1B1 IHC staining (one-way ANOVA). Scale bar = 100 μm. **E–F** LNCaP and LNCaP-abl cells were treated with 20 μM bicalutamide for 3 days. CYP1B1 expression was examined by western blotting (**E**), and intracellular 4-OHE2 concentration was examined by HPLC–MS (**F**). **G**, The cell viability of CYP1B1-overexpressing and control LNCaP cells treated with bicalutamide was determined by MTT assay (t-test). **H** The cell viability of CYP1B1 knockdown and control LNCaP-abl cells treated with bicalutamide was determined by MTT assay (t-test). **I** Left: Cell invasion capability was assayed in CYP1B1-overexpressing or control LNCaP cells after bicalutamide treatment. Right: Quantitative result of the left panel(t-test). Scale bar = 500 μm. **J** Left: Cell invasion capability was assayed in CYP1B1 knockdown or control PC3 cells (t-test). Right: Quantitative result of the left panel. Scale bar = 500 μm. **K** Left: Representative IF staining of VIM (red) and CYP1B1 (green) in LNCaP and PC3 cells. Right: Quantitative result of the left panel. Scale bar = 100 μm. AS, androgen sensitivity; AI, androgen insensitivity; Bic, bicalutamide; VIM, vimentin. All values represent the means ± SD from three independent experiments. *p < 0.05, **p < 0.01, **p < 0.001

We evaluated CYP1B1 expression in publicly available gene expression databases, and the results showed that *CYP1B1* expression was significantly higher in CRPC than in primary PCa (Fig. 1A). In addition, qRT-PCR and western blot assays showed that androgen-independent cell lines (LNCaP-abl and PC3) displayed higher levels of CYP1B1 than the androgen-dependent cell line (LNCaP) (Fig. 1B). The HPLC–MS assay displayed that PC3 cells exhibited a higher 4-OHE2 concentration than LNCaP cells (Fig. 1C), and CYP1B1 knockdown in PC3 cells decreased the intracellular 4-OHE2 concentration (Additional file 2: Fig. S4–S5). Moreover, an immunohistochemical analysis was carried out to examine the protein expression of CYP1B1 in benign prostate tissues, PCa tissues (Gleason score ≤ 6 and Gleason score 8–10) and CRPC tissues. In benign prostate tissues, we detected CYP1B1 staining in some epithelial cells, especially in basal epithelial cells. Notably, tumors with a high Gleason score (Gleason 8–10) showed stronger CYP1B1 staining. Compared with the PCa tissues, CRPC tissues displayed higher expression levels of CYP1B1 (Fig. 1D). These results suggest that increased CYP1B1 expression has a positive association with CRPC progression.

To explore whether enhanced CYP1B1 levels promote androgen-independence of PCa cells, we cultured LNCaP and LNCaP-abl cells and treated them with bicalutamide, an androgen receptor blocker usually used for ADT, to simulate the CRPC process. We detected a significant increase in CYP1B1 expression and 4-OHE2 concentration in LNCaP and LNCaP-abl cells after bicalutamide treatment (Fig. 1E, F). Increasing metastasis and decreasing cell sensitivity to ADT are the main contributors to CRPC development. We found that CYP1B1-overexpressing stable cells had higher cell viability under culture conditions with bicalutamide, while CYP1B1 knockdown LNCaP-abl cells showed attenuated cell viability upon bicalutamide treatment (Fig. 1G, H, Additional file 2: Fig. S4). Furthermore, CYP1B1 overexpression significantly increased LNCaP cell invasion upon bicalutamide treatment, while knockdown of CYP1B1 in an androgen-independent PCa cell line (PC3) markedly decreased cell invasiveness (Fig. 1I, J). The IF assay displayed a higher rate of CYP1B1/VIM coexpression in the androgen-independent PCa cell line (PC3) than in the androgen-dependent PCa cell line (LNCaP) (Fig. 1K). Together, these results indicate that elevated CYP1B1 expression could decrease bicalutamide sensitivity in PCa cells.

### CYP1B1 knockdown attenuates PCSC characteristics in CRPC cells

**Fig. 2:**
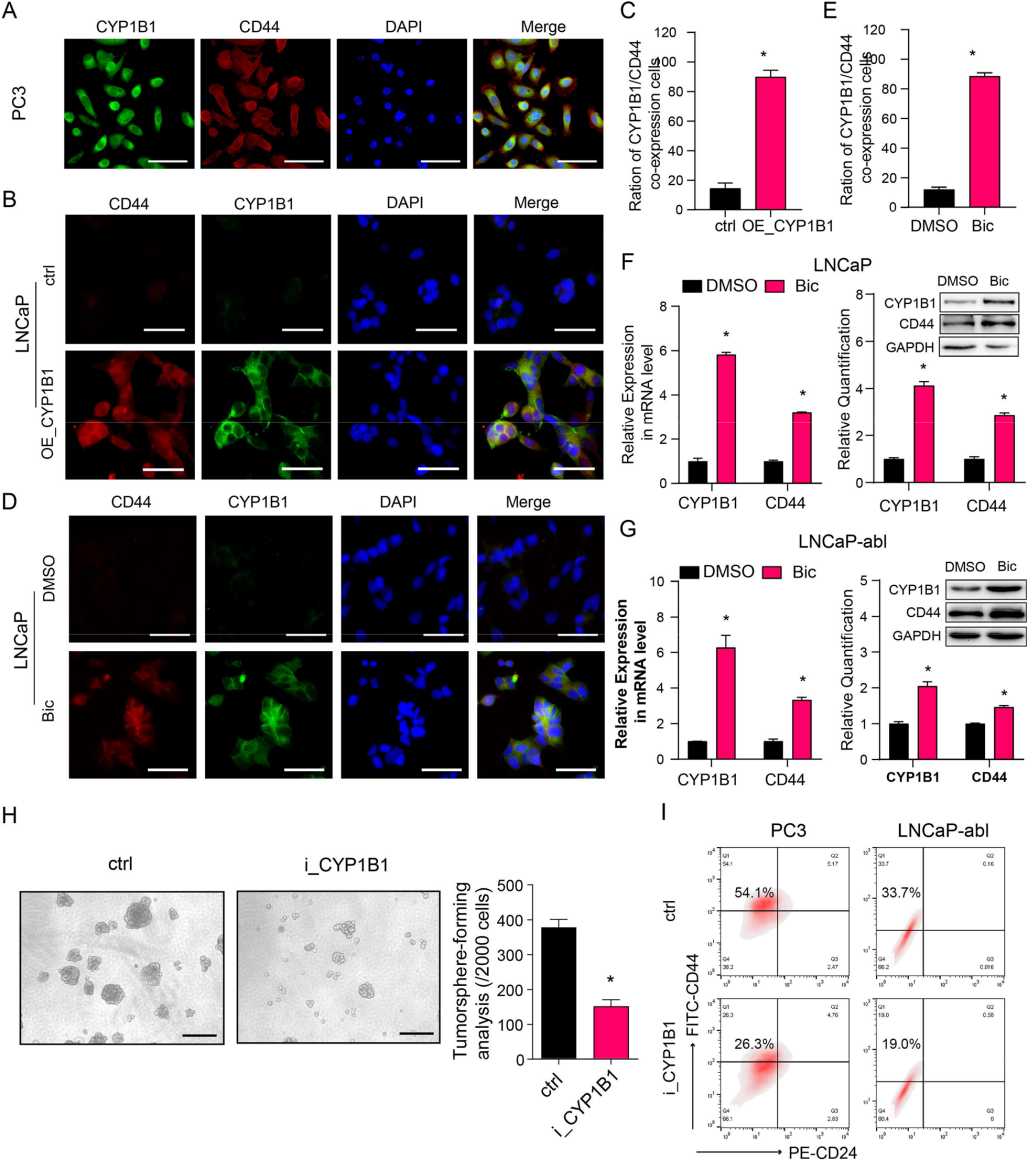
CYP1B1 is critical to maintaining the PCSC subpopulation in PCa. **A** Representative IF staining of CYP1B1 (green) and CD44 (red) in PC3 cells. Scale bar = 50 μm. **B–E** Colocalization of CD44 and CYP1B1 (t-test). **B**, **D** Representative IF staining of CD44 (red) and CYP1B1 (green) in LNCaP cells after the indicated treatment. Scale bar = 50 μm. **D** Quantification of (**B**). **E** Quantification of (**C**). **F**, **G** mRNA (left) and protein (right) levels of CYP1B1 and CD44 in bicalutamide- or DMSO-treated PCa cells (t-test). **H** Representative image and quantification of tumorsphere formation in CYP1B1 knockdown or control LNCaP-abl cells (t-test). Scale bar = 100 μm. **I** A representative flow cytometric analysis of the proportion of PCSCs (CD44^+^/CD24^−^) in CYP1B1 knockdown or control PCa cells. All values represent the means ± SD from three independent experiments. *p < 0.05

To further examine the effect of CYP1B1 on PCa progression, we analyzed CYP1B1^High^ (n = 50) and CYP1B1^Low^ (n = 50) available prostate adenocarcinoma (PRAD) samples in the TCGA dataset. The gene expression profiles of samples with different CYP1B1 expression levels were hierarchically clustered and visualized in a heatmap using Morpheus. Some basal epithelial cell markers and cancer stem-like cell markers had significantly higher expression levels in CYP1B1^High^ samples than CYP1B1^Low^ samples (Additional file 2: Fig. S6A), and overexpressing CYP1B1 in LNCaP cells significantly upregulated the expression of basal epithelial cell markers *TP63*, *KRT5*, and *KRT14*, as well as some stemness-associated markers, including *CD44*, *KIT*, *MET*, *MAML2*, *SOX2*, *OCT4*, and *ABCG2* (Additional file 2: Fig. S6B). CD44, a PCSC marker, is known to promote CRPC. Using an IF assay, we found better colocalization of CYP1B1 and CD44 in CRPC tissues and PC3 cells (Fig. 2A, Additional file 2: Fig. S6C). Consistently, we detected increased numbers of LNCaP cells expressing both CYP1B1 and CD44 after CYP1B1 overexpression or bicalutamide treatment (Fig. 2B–E). In addition, CYP1B1 and CD44 expression levels were significantly increased after bicalutamide treatment in LNCaP and LNCaP-abl cells (Fig. 2F, G), implying that CD44^+^ PCSCs with high CYP1B1 expression were increased during CRPC progression.

To evaluate the expression of CYP1B1 in PCSCs, we performed serum-free suspension culture to enrich PCSCs. We found that the expression of *CYP1B1*, together with the stem-associated markers *CD44*, *SOX2*, and *OCT4*, was significantly elevated in PCSCs compared with adherent monolayers (Additional file 2: Fig. S6D). CYP1B1 knockdown significantly suppressed tumorsphere formation (Fig. 2H). Flow cytometry assays showed that the CD44^+^/CD24^-^ subpopulation was significantly decreased in CYP1B1 knockdown cells compared with control cells (Fig. 2I). In addition, knockdown of CYP1B1 in PC3 and LNCaP-abl cells decreased the expression of basal markers and stemness-associated genes (Additional file 2: Fig. S6E-F).

### CYP1B1 upregulates IL6-STAT3 signaling by inducing IL6 expression

**Fig. 3:**
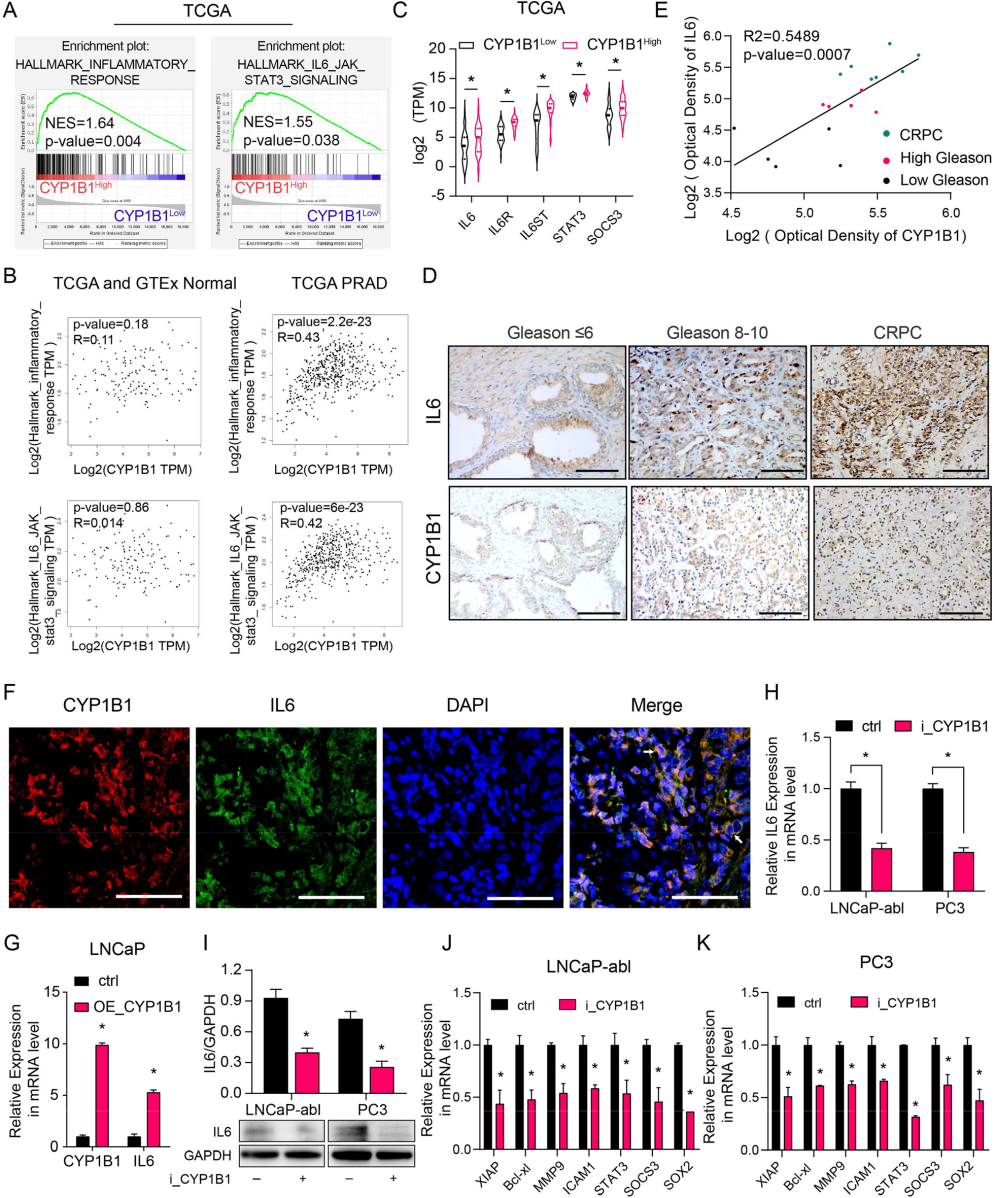
CYP1B1 regulates the IL6-STAT3 signaling by inducing IL6 expression. **A** Gene signatures associated with the inflammatory response and IL6-jak-stat3 signaling were analyzed by GSEA in CYP1B1^High^ and CYP1B1^low^ PRAD samples from the TCGA. **B** Correlation analysis of CYP1B1, IL6-JAK-STAT3 signaling gene set and inflammatory response gene set in normal prostate and PRAD patients from TCGA (TCGA and GTEx normal, n = 152; PRAD, n = 498). **C**, Mean mRNA levels of *IL6*, *IL6R*, *IL6ST*, *STAT3* and *SOCS3* for CYP1B1^High^ samples versus CYP1B1^low^ samples in the PRAD dataset from TCGA. **D–E** Representative images of CYP1B1 and IL6 expression measured by IHC in PCa samples with low and high Gleason scores and in CRPC tissues (**D)**. Scale bar = 100 μm. **E** The average optical densities of CYP1B1 and IL6 were calculated, followed by correlation analysis of CYP1B1 and IL6 expression in primary PCa and CRPC tissues. **F** IF staining of CYP1B1 (red) and IL6 (green) in PCa tissue. Scale bar = 50 μm. **G–I** IL6 expression in CYP1B1-overexpressing LNCaP cells (**G**) and CYP1B1 knockdown PCa cells (**H**, **I**) (t-test). **J**, **K** Relative expression of the indicated IL6-STAT3 pathway downstream genes in CYP1B1 knockdown LNCaP-abl (**J**) and PC3 (**K**) cells by qRT-PCR analysis (t-test). All values represent the means ± SD from three independent experiments. *p < 0.05

To further define the pathway modulated by CYP1B1 in PCa, we performed GSEA between CYP1B1^High^ and CYP1B1^Low^ PRAD samples from the TCGA dataset. The results showed that the IL6-STAT3 pathway and inflammatory response gene signatures were enriched in CYP1B1^High^ samples (Fig. 3A). Correlation analysis displayed that CYP1B1 expression was positively correlated with the inflammatory response and IL6-STAT3 pathway (Fig. 3B). Additionally, the mean mRNA expression analysis showed the *IL6*, *IL6R*, *IL6ST*, *STAT3*, and *SOCS3* expression were aberrantly upregulated in CYP1B1^High^ samples (Fig. 3C).

IL6 is known to play a decisive role in the IL6-STAT3 pathway. Therefore, we speculated that CYP1B1 regulates IL6 expression to stimulate the IL6-STAT3 pathway. PCSCs harbored higher levels of IL6, similar to CYP1B1 expression in PCSCs (Additional file 2: Fig. S7). Immunohistochemical analysis was carried out to examine the protein expression of CYP1B1 and IL6 in primary PCa (Gleason score ≤ 6 and Gleason score 8–10) and CRPC tissues. The results showed that CRPC tissues expressed significantly higher levels of CYP1B1 and IL6 than primary PCa tissues (Fig. 3D, E). Moreover, CYP1B1 and IL6 were highly coexpressed in PCa tissues and PC3 cells, and IL6 and CD44 were coexpressed in PC3 cells (Fig. 3F, Additional file 2: S8–S9). Furthermore, CYP1B1 overexpression led to an increase in the expression of IL6 in LNCaP cells (Fig. 3G). In contrast, knockdown of CYP1B1 expression in LNCaP-abl and PC3 cells significantly reduced the expression of IL6 and its downstream genes (Fig. 3H–K).

### CYP1B1 enhances PCSC characteristics by 4-OHE2-induced IL-6-STAT3 pathway activation

**Fig. 4:**
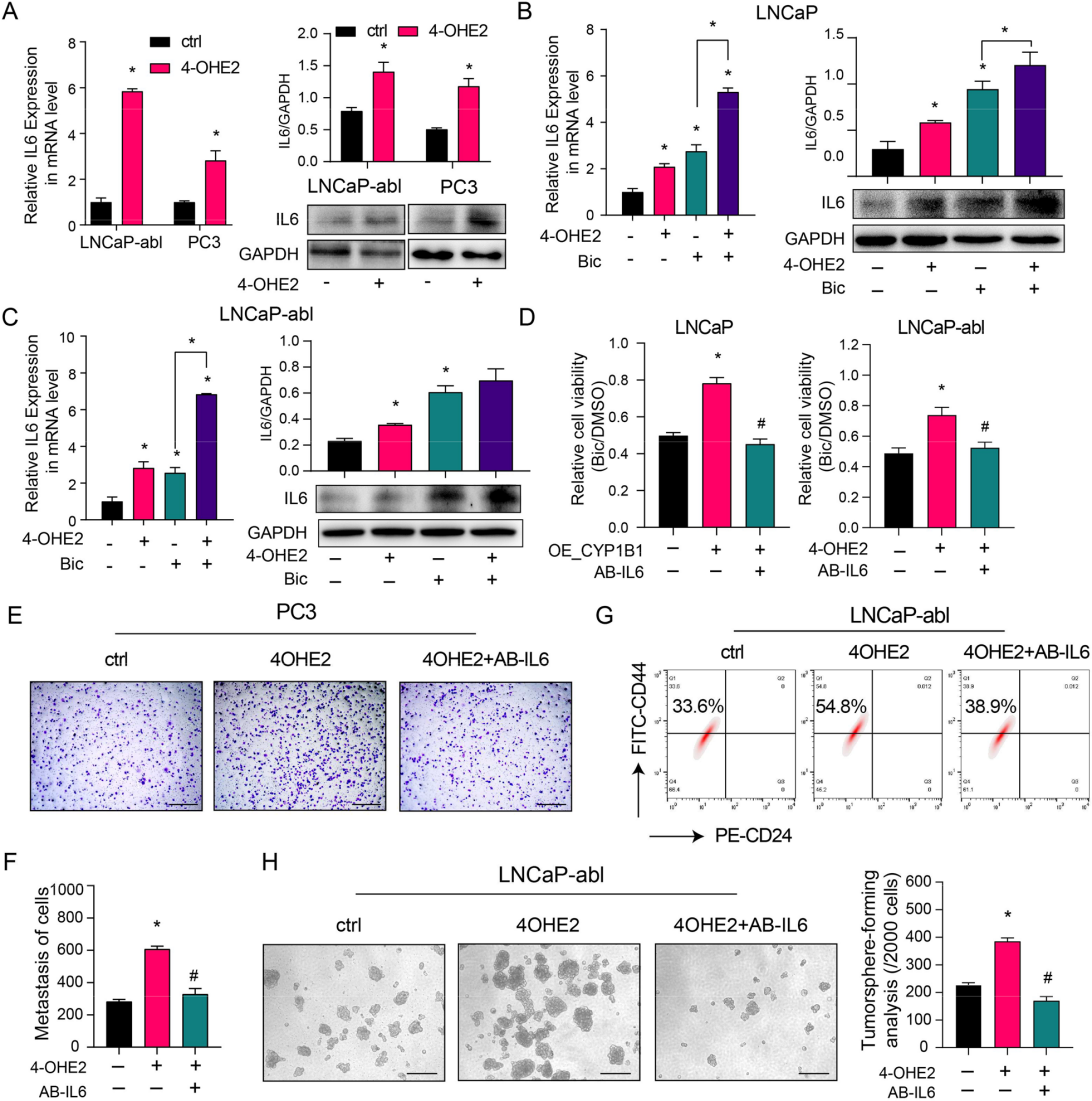
4-OHE2 promotes PCSC characteristics by upregulating IL6 expression. **A** The mRNA (left) and protein (right) levels of IL6 in LNCaP-abl and PC3 cells treated with 4-OHE2 (t-test). **B–C** The mRNA (left) and protein (right) levels of IL6 in LNCaP (**B**) and LNCaP-abl (**C**) cells after the indicated treatments (one-way ANOVA). **D** Cell viability was assayed in LNCaP (left) and LNCaP-abl (right) cells after the indicated treatments (one-way ANOVA). **E–F**, (**E**) The cell invasion capability was assayed in PC3 cells after the indicated treatments. (**F**) Quantitative result of the (**E**) (one-way ANOVA). Scale bar = 500 μm. **G** A representative flow cytometric analysis of the CD44^+^/CD24^−^ subpopulation of LNCaP-abl cells treated with 4-OHE2 and/or AB-IL6. **H** Representative image (left) and quantification (right) of tumorsphere formation assay from LNCaP-abl cells treated with 4-OHE2 and/or AB-IL6. Scale bar = 100 μm (one-way ANOVA). Bic, bicalutamide; AB-IL6, IL6 neutralizing antibody. All values represent the means ± SD from three independent experiments. *p < 0.05 versus ctrl. #*p* < 0.05 versus 4-OHE2 or OE_CYP1B1

To determine the effect of 4-OHE2 on IL6 expression, we observed dramatically increased IL6 expression levels after short-term 4-OHE2 treatment in PC3 and LNCaP-abl cells, accompanied by upregulation of IL6 downstream gene expression (Fig. 4A, Additional file 2: Fig. S10). We also detected that 4-OHE2 significantly upregulated IL6 expression after bicalutamide treatment in both LNCaP and LNCaP-abl cells (Fig. 4B, C). In addition, IL6 neutralizing antibody (AB-IL6) treatment decreased the effect of 4-OHE2 on the mRNA expression of IL6-STAT3 signaling target genes (Additional file 2: Fig. S11). Furthermore, AB-IL6 abrogated 4-OHE2-induced cell invasion, bicalutamide resistance, tumorsphere formation efficiency, and the number of the CD44^+^/CD24^−^ PCSCs subpopulation (Fig. 4D–H).

### 4-OHE2 directly upregulates IL6 expression through ERα-mediated transcriptional activity

**Fig. 5:**
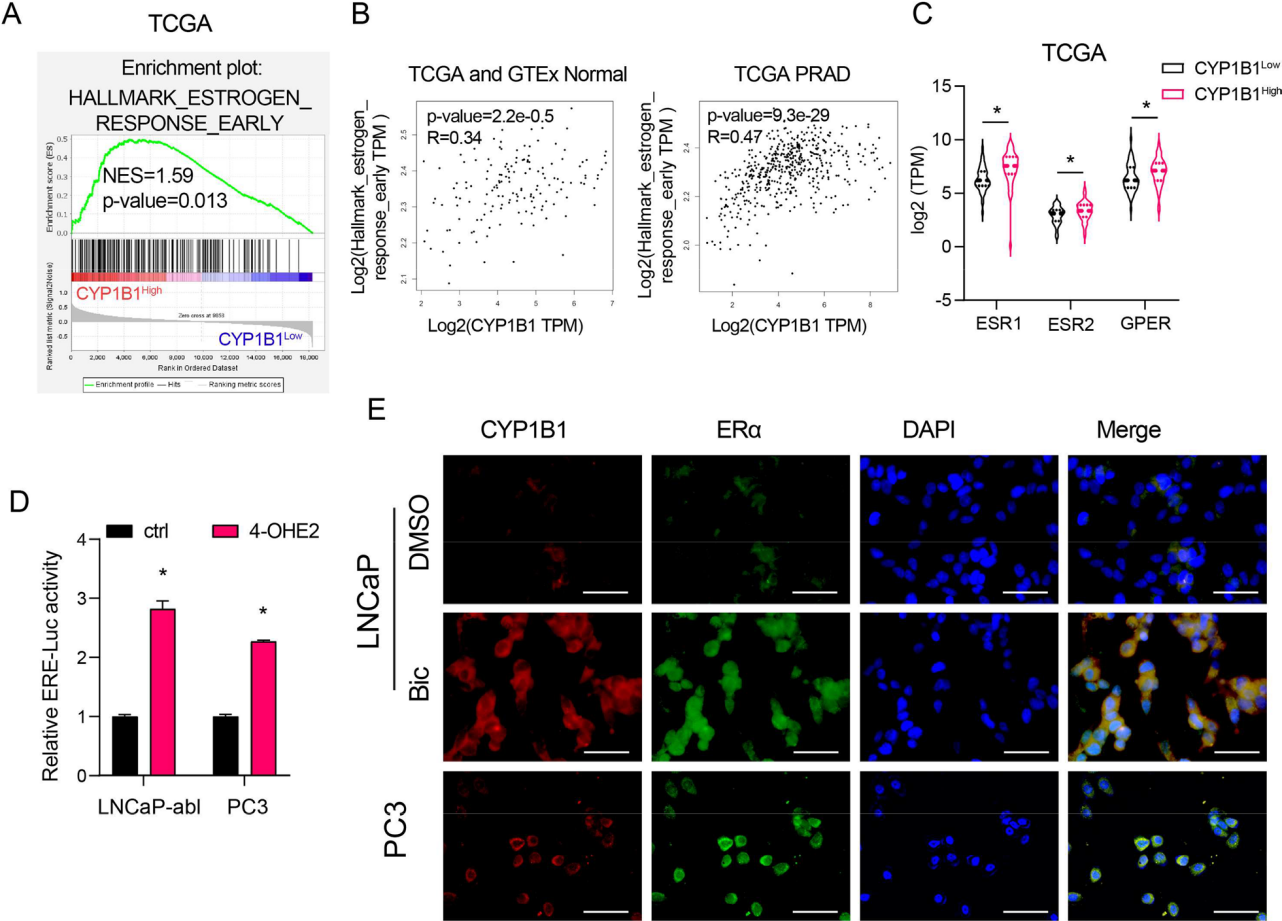
4-OHE2 enhances ER transcriptional activity. **A** Identification of gene signatures related to an early estrogen response by GSEA in CYP1B1^High^ and CYP1B1^low^ PRAD samples from the TCAG. **B** Correlation analysis of CYP1B1 and the early estrogen response gene set in normal prostate tissues and PRAD tissues from TCGA (TCGA and GTEx normal, n = 152, PRAD, n = 498). **C** Mean mRNA levels of ESR1, ESR2 and GPER for CYP1B1^High^ sample versus CYP1B1^Low^ samples in the PRAD dataset from the TCGA (t-test). **D** Luciferase reporter assays in LNCaP-abl and PC3 cells transfected with estrogen receptor promoter reporter (ERE-LUC) after 4-OHE2 treatment (t-test). **E** Representative IF staining of CYP1B1 (red) and ERα (green) in LNCaP and PC3 cells after the indicated treatments. Scale bar = 50 μm. Bic, bicalutamide. PRAD, prostate adenocarcinoma. All values represent the means ± SD from three independent experiments. *p < 0.05

GSEA and correlation analysis results revealed that CYP1B1 expression was positively correlated with signatures representative of the early estrogen response (Fig. 5A, B). We also discovered that the mean expression levels of estrogen receptors (ERα, ERβ and GPR30) in CYP1B1^High^ PRAD samples were dramatically higher than those in CYP1B1^Low^ PRAD samples, especially ERα (Fig. 5C). 4-OHE2 treatment significantly increased ERE promoter activity (Fig. 5D). IF assays showed that the number of cells expressing both CYP1B1 and ERα was increased in LNCaP cells after bicalutamide treatment and that CYP1B1 and ERα were coexpressed in PC3 cells (Fig. 5E). We also found that CD44 and ERα were coexpressed in PC3 cells (Additional file 2: Fig. S12). These results indicated that 4-OHE2 promotes ERα transcriptional activity in PCa cells.

**Fig. 6:**
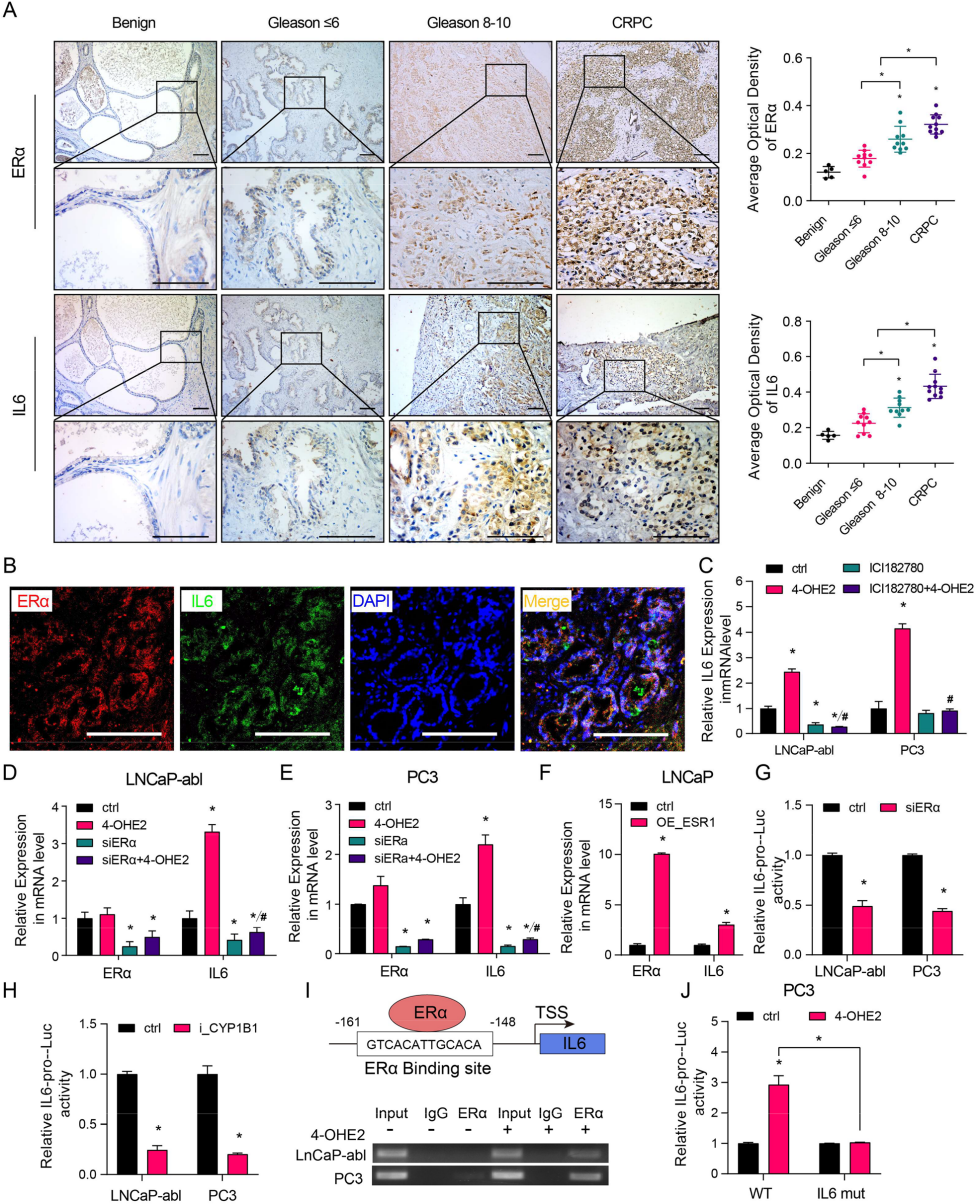
4-OHE2 upregulates IL6 expression via ERα. **A** Left: Representative IHC staining of ERα and IL6 protein in benign prostate tissues, primary PCa and CRPC tissues. Right: Quantification of the average optical density of ERα and IL6 by IHC staining. Scale bar = 100 μm (one-way ANOVA). **B** IF staining of ERα (red) and IL6 (green) in PCa tissue. Scale bar = 100 μm. **C** The mRNA level of IL6 in LNCaP-abl and PC3 cells after the indicated treatments (one-way ANOVA). **D**–**E** The mRNA level of IL6 was assayed in ERα knockdown or control LNCaP-abl (**D**) and PC3 (**E**) cells after 4-OHE2 treatment (one-way ANOVA). **F** qRT-PCR analysis of IL6 levels in ERα-overexpressing or control LNCaP cells (t-test). **G–H** Luciferase reporter assays in LNCaP-abl and PC3 cells transfected with IL6 promoter reporter (IL6-pro) together with ERα knockdown (**G**) or CYP1B1 knockdown (**H**) (t-test). **I** Top: Predicted ERα-binding site in the proximal promoter region of IL6. Bottom: PC3 and LNCaP-abl cells treated with or without 10 nM 4-OHE2 were subjected to ChIP analysis using an anti-ERα antibody or isotype-matched IgG control. **J** Luciferase reporter assays in PC3 cells transfected with the IL6 promoter containing wild-type (WT) or mutated ERα-binding site (IL6 mut) together with DMSO or 4-OHE2 treatment (t-test). All values represent the means ± SD from three independent experiments. *p < 0.05 versus ctrl. #*p* < 0.05 versus 4-OHE2

Immunohistochemical analysis was carried out to examine the protein expression of ERα and IL6 in benign prostate tissues, primary PCa (Gleason score ≤ 6 and Gleason score 8–10) and CRPC tissues. The results showed that ERα and IL6 had similar expression trends in PCa tissues, and CRPC tissues expressed significantly higher levels of ERα and IL6 than primary PCa tissues (Fig. 6A). The IF assay showed that ERα and IL6 were coexpressed in PCa cells (Fig. 6B). To explore whether 4-OHE2 upregulates IL6 levels via ERα, PC3 and LNCaP-abl cells were treated with the pure ER antagonist ICI182780 before being cultured with 4-OHE2. Competitive inhibition of 4-OHE2 binding to ERα exerted by ICI182780 completely prevented the effect of 4-OHE2 on IL6 expression in PC3 and LNCaP-abl cells (Fig. 6C). Furthermore, ERα knockdown in PC3 and LNCaP-abl cells noticeably suppressed IL6 expression, and 4-OHE2 did not restore IL6 expression (Fig. 6D, E). In contrast, ERα overexpression enhanced the IL6 expression in LNCaP cells (Fig. 6F).

To determine the mechanism underlying ERα-mediated IL6 expression, we detected a potential ERα binding site in the IL6 promoter by using the JASPAR database. The Dual-Luciferase® Reporter (DLR™) assay revealed that ERα knockdown reduced IL6 promoter activity, which was similar to the finding in CYP1B1 knockdown cells (Fig. 6G, H). The competency for ER binding was further examined using a ChIP assay. ERα was recruited to the ERα-binding site of the IL6 promoter in PC3 and LNCaP-abl cells, and the effect was enhanced after exposure to 4-OHE2 (Fig. 6I). Moreover, we constructed IL6 mutation promoter plasmids to determine the effect of 4-OHE2/ERα on IL6 transcription. A luciferase reporter assay displayed that 4-OHE2 increased IL6 promoter activity, whereas mutation of the ERα-binding site abrogated the effect of 4-OHE2 treatment (Fig. 6J).

## Discussion

It has been reported that the expression of CYP1B1 is significantly increased in hormone-related cancers, including breast cancer, endometrial cancer, ovarian cancer, and PCa [19, 26, 40]. PCa patients with high CYP1B1 expression have lower survival rates [27]. Here, we found that the expression of CYP1B1 was positively correlated with the Gleason score of PCa, with the highest expression in CRPC tissues. Compared with androgen-dependent PCa cells (LNCaP), androgen-independent PCa cells (LNCaP-abl, PC3) had higher levels of CYP1B1. It has been reported that the high expression of CYP1B1 promotes the resistance of ovarian cancer cells and PCa cells to docetaxel [41]. We found that CYP1B1 reduced the sensitivity of PCa cells to bicalutamide. Overexpression of CYP1B1 decreased bicalutamide sensitivity, and CYP1B1 knockdown increased cell sensitivity to bicalutamide, suggesting that increased CYP1B1 expression in PCa cells could promote CRPC progression.

In human breast epithelial cells, 4-OHE2 has been reported to promote tumorigenesis by generating potentially mutagenic free radicals through metabolic redox cycling among its quinone, semiquinone and hydroquinone forms, and this process is not dependent on the estrogen receptor signaling pathway [42, 43]. Other studies have shown that 4-OHE2 could exert xenoestrogen effects in breast cancer cells by binding to ERα [44]. Knockdown of catechol-O-methyltransferase (COMT) in PCa cells, which catalyzes the conversion of 4-OHE2 to 2-methoxy-estradiol, promotes the proliferation of ERα^+^ PC3 cells but inhibits the proliferation of ERα^−^ Du145 cells [45]. Based on the GSEA of the PCa gene expression profiles in TCGA, we found that CYP1B1 expression was positively correlated with the early estrogen response gene set. Compared with CYP1B1^Low^ PCa patients, CYP1B1^High^ PCa patients had higher ERα levels. Moreover, CYP1B1 and ERα were coexpressed in PC3 cells. In LNCaP cells, bicalutamide treatment significantly enriched cells expressing CYP1B1 and ERα, and increased intracellular 4-OHE2 levels. 4-OHE2 significantly increased ERα transcriptional activity in PCa cell lines. These data suggest that 4-OHE2 can play a role in regulating ERα in CRPC.

The activation of PCSCs is related to the transition from androgen sensitivity to castration resistance [46, 47]. Our previous studies have shown that the elevated endogenous estradiol concentration is related to the enrichment of CD44^+^ PCSCs in CRPC progression [34]. In inflammatory breast cancer, the CD44^+^/CD24^−/low^ cancer stem cell subset harbors higher levels of CYP1B1 [48], and 4-OHE2 treatment induces anchorage-independent growth of mammary epithelial cells [23]. However, the expression of CYP1B1 (an enzyme that catalyzes estradiol to 4-OHE2) and the role of 4-OHE2 in PCSCs have not been reported. Here, we found that CYP1B1 was highly expressed in CD44^+^ cells of CRPC tissues and in androgen-independent PCa cell lines. In LNCaP cells, the upregulation of CYP1B1 expression after bicalutamide treatment is associated with the enrichment of CD44^+^ subsets. CD44^+^/CD24^−^ PCSCs, enriched from LNCaP-abl cells, exhibited higher CYP1B1 expression than adherent cells. Besides, CYP1B1 overexpression and 4-OHE2 treatment upregulated the expression of stemness-associated genes, while CYP1B1 knockdown significantly reduced the proportion of CD44^+^/CD24^−^ PCSCs and the tumorsphere formation ability in PCa cell lines. These results indicate that 4-OHE2, catalyzed by CYP1B1, can promote PCSC characteristics.

We further explored the mechanism by which 4-OHE2 regulates PCSC characteristics. Studies have shown that CYP1B1 inhibitors can block the activation of multiple proinflammatory signaling pathways in tumor cells and in the tumor microenvironment [49]. The IL6-STAT3 signaling pathway is essential in the inflammatory response of tumor tissues, and the IL6- STAT3 signaling pathway has been reported to promote the enrichment of PCSCs in PCa tissues treated with ADT [32]. Based on GSEA of the PCa gene expression profiles in TCGA, we found that CYP1B1 expression was positively correlated with signatures representative of the inflammatory response and IL6-JAK-STAT3 signaling. CYP1B1^High^ prostate adenocarcinoma patients displayed higher IL6 levels than CYP1B1^Low^ prostate adenocarcinoma patients, and coexpression of CYP1B1 and IL6 was found in PCa tissues and PCa cell lines. In PCa cell lines, 4-OHE2 significantly upregulated IL6 expression, increased the proportion of CD44^+^/CD24^−^ PCSCs, increased tumorsphere formation ability, and decreased bicalutamide sensitivity, while IL6 neutralizing antibodies reversed these effects. Studies have shown that treatment of mouse prostate tissue with estradiol can induce inflammation, and the concentrations of endogenous estradiol and 4-OHE2 are significantly increased under these conditions [50]. Estrogen has been reported to induce the occurrence of PCa by activating the inflammatory response [51]. We found that 4-OHE2 promotes PCSC characteristics by activating the inflammation-associated IL6 expression, which provides new insights into the mechanisms by which estrogen promotes CRPC progression.

Studies have shown that estradiol and its receptor are involved in regulating IL6 expression and have tissue cell specificity. In triple-negative breast cancer, G protein-coupled estrogen receptor (GPER) inhibits cell invasion and angiogenesis by suppressing NF-κB/IL-6 signaling [52]. In non-small cell lung cancer, estradiol upregulates the expression of IL-6 under the mediation of ERβ [53]. It has been reported that ERα can upregulate IL6 expression in biliary epithelial cells [54]. We found that the ER antagonist ICI182780 or siERα inhibited the upregulation of IL6 expression induced by 4-OHE2 in PCa cells. ChIP experiments showed that 4-OHE2 promoted ERα binding to the estrogen response element (ERE) in the IL6 promoter. These findings suggest that 4-OHE2 directly upregulates the expression of IL6 by regulating ERα activity.

## Conclusion

Our study reveals that the increased endogenous 4-OHE2 concentration catalyzed by high levels of CYP1B1 promotes PCSC characteristics, which is one of the main contributors to CRPC progression during ADT. Moreover, ERα-mediated 4-OHE2 effects upregulate the expression of IL6 via the binding and transcriptional activation of the *IL6* promoter, which further enhances IL6-STAT3 pathway activation. These findings provide new insights into the molecular mechanism underlying CRPC progression, implicating and revealing that CYP1B1 may be a new target for CRPC therapeutic intervention.

## Supplementary Information

**Additional file 1**. Additional protocols tables. Tables S1-S3 show additional data related to the methods of the study. **Table S1.** Primers used for qRT-PCR. **Table S2.** Primary antibodies used in the western blotting and IF assays. **Table S3.** Primers used for plasmid construction.

**Additional file 2**. Additional results figures. Figures S1-S12 show additional data related to the results shown in the main figures. **Figure S1. The plasmid construction process and plasmid maps**. A, pcDNA3.1( +)-CYP1B1 plasmid construction diagram. B-D, Plasmid maps of pcDNA3.1( +)-CYP1B1, CRISPRi-CYP1B1 and CRISPRi-Lac. **Figure S2. Transfection efficiency/cytotoxicity of the plasmids**. pcDNA3.1( +) plasmids, which expresses 3 × flag-tagged peptide, were transfected into LNCaP cells. The transfection efficiency (A) and cytotoxicity (B) of the plasmids were assessed. Scale Bar = 50 μm. **Figure S3.** Selected ion monitoring (SIM) chromatograms of the 4-OHE2 standard. E2 was dissolved in methanol. The monitored ion in the mass spectrum had a mass/charge ratio of 755 m/z and a retention time of 6.95 min. **Figure S4.** Validation of CYP1B1 silencing efficiency in LNCaP-abl and PC3 cells and CYP1B1 overexpression efficiency in LNCaP cells by western blotting. **Figure S5.** HPLC–MS assay results showing the concentration of intracellular 4-OHE2 in CYP1B1 knockdown or control PC3 cells (t-test). *p < 0.05. **Figure S6. The effect of CYP1B1 on the expression of stemness-associated genes**. **A**, Characteristics of differential gene expression profiles of CYP1B1^High^ and CYP1B1^Low^ PRAD samples from the TCGA. **B**, The expression of some basal cell markers and stemness-associated genes was verified by qRT-PCR in CYP1B1-overexpressing LNCaP cells compared with control LNCaP cells (t-test). **C**, Representative IF staining of CD44 (red) and CYP1B1 (green) in CRPC tissue. Scale bar = 50 μm. **D**, The expression of CYP1B1 and stemness-associated genes was tested in spheroid cells (PCSCs) and adherent cells (Bulk) by qRT-PCR (t-test). **E–F**, Relative mRNA levels of some stemness-associated genes were verified by qRT-PCR in PC3 (**E**) and LNCaP-abl (**F**) cells after CYP1B1 knockdown (t-test). All values represent the means ± SD from three independent experiments. *p < 0.05. **Figure S7.** *IL6* expression was tested in spheroid cells (PCSCs) and adherent cells (Bulk) by qRT-PCR. All values represent the means ± SD from three independent experiments. *p < 0.05. **Figure S8.** IF staining of CYP1B1 (red) and IL6 (green) in PC3 cells. Scale bar = 50 μm. **Figure S9.** IF staining of CD44 (red) and IL6 (green) in PC3 cells. Scale bar = 50 μm. **Figure S10.** qRT-PCR analysis of the indicated IL6-STAT3 target genes in LNCaP-abl (**A**) and PC3 (**B**) cells treated with 4-OHE2 or IL6 (one-way ANOVA). *p < 0.05. **Figure S11.** qRT-PCR analysis of the indicated IL6-STAT3 target genes in LNCaP-abl and PC3 cells treated with 4-OHE2 and/or AB-IL6 (one-way ANOVA). *p < 0.05. **Figure S12.** IF staining of ERα (red) and CD44 (green) in PC3 cells.

## Data Availability

Additional data during this study are available in the Methods sections.

## Abbreviations

IL6: Interleukin 6
STAT3: Signal transducer and activator of transcription 3;
E2: 17β-Estradiol
4-OHE2: 4-Hydroxy-17β-estradiol. ERα: Estrogen receptor-α
Bic: Bicalutamide
ADT: Androgen deprivation therapy
PCa: Prostate cancer
CRPC: Castration-resistant prostate cancer
PCSC: Prostate cancer stem cell
PRAD: Prostate adenocarcinoma
TCGA: The Cancer Genome Atlas
ChIP: Chromatin Immunoprecipitation
IF: Immunofluorescence
IHC: Immunohistochemistry
RT-PCR: Quantitative real-time reverse-transcription-polymerase chain reaction
HPLC–MS: High–performance liquid chromatography-mass spectrometry
