# Supplementary material for: CYP1B1-catalyzed 4-OHE2 promotes the castration resistance of prostate cancer stem cells by estrogen receptor α-mediated IL6 activation
Source: Cell Commun Signal. 2022 Mar 15;20:31. doi: 10.1186/s12964-021-00807-x (PMC8922936; doi:10.1186/s12964-021-00807-x)
Supplement: Supplementary file 2 — Additional file 1. Additional protocols tables. Tables S1-S3 show additional data related to the methods of the study. Table S1. Primers used for qRT-PCR. Table S2. Primary antibodies used in the western blotting and IF assays. Table S3. Primers used for plasmid construction. [file 12964_2021_807_MOESM2_ESM.docx]

**Additional file 1.**

**Table S1**. Primers used for qRT-PCR.

| Genes | Forward Primer (5’-3’) | Reverse Primer (5’-3’) |
| --- | --- | --- |
| CYP1B1 | GTACCGGCCACTATCACTGACA | CACATCAGGATACCTGGTGAAGAG |
| ERα | TGGGCTTACTGACCAACCTG | CCTGATCATGGAGGGTCAAA |
| IL6 | ACTCACCTCTTCAGAACGAATTG | CCATCTTTGGAAGGTTCAGGTTG |
| HPRT | TGACACTGGCAAAACAATGCA | GGTCCTTTTCACCAGCAAGCT |
| TP63 | GGTTGGCAAAATCCTGGAG | GGTTCGTGTACTGTGGCTCA |
| KRT5 | TGGAGATCGCCACTTACCGCAAG | TACCTCCGGCAAGACCTCCACCG |
| KRT14 | GACTTCCGGACCAAGTTTGA | CTTGAGGCTCTCAATCTGC |
| MET | ATCAGAGGGTCGCTTCATGC | TCACTTCTGGAGACACTGGA |
| MAML2 | AAGCGACCCAATGGCTTTGT | GAGTGTTAGTCTTTCGCAGGG |
| CD44 | TCCAACACCTCCCAGTATG | TTCTGGACATAGCGGGTG |
| SOX2 | TGGCGAACCATCTCTGTGGT | CCAACGGTGTCAACCTGCAT |
| OCT4 | GTGGAGGAAGCTGACAACAA | ATTCTCCAGGTTGCCTCTCA |
| ABCG2 | CGCGACCTGCCAATTTCAAA | CAACCTTGGAGTCTGCCACT |
| IL6R | CATGTGCGTCGCCAGTAGT | AGCTCAAACCGTAGTCTGTAGA |
| IL6ST | GGCAGCATACACAGATGAAGG | CAAACAGGCACGACTATGGC |
| STAT3 | CAGCAGCTTGACACACGGTA | AAACACCAAAGTGGCATGTGA |
| SOCS3 | CATGGTCACCCACAGCAAGT | CCAGCTGGTACTCGCTCTTG |
| XIAP | AATAGTGCCACGCAGTCTACA | CAGATGGCCTGTCTAAGGCAA |
| Bcl-xl | GAGCTGGTGGTTGACTTTCTC | TCCATCTCCGATTCAGTCCCT |
| MMP9 | GGTGATTGACGACGCCTTTG | GGACCACAACTCGTCATCGT |
| ICAM1 | GTATGAACTGAGCAATGTGCAAG | GTTCCACCCGTTCTGGAGTC |

**Table S2.** Primary antibodies used in the western blotting and IF assays.

| Primary antibodies | Corporation | Apply | Dilution ratio |
| --- | --- | --- | --- |
| Mouse GAPDH | KANG CHEN, KC-5G4 | WB | 1:5000 |
| Rabbit anti-CYP1B1 | Abcam, ab32649 | IF | 1:200 |
| Mouse anti-CYP1B1 | Santa, sc-374228 | IF | 1:200 |
|  |  | WB | 1:1000 |
| Rabbit anti-IL6 | Abcam, ab6672 | IF | 1:100 |
|  |  | WB | 1:1000 |
| Mouse anti-KRT5 | Santa, sc-32721 | IF | 1:200 |
| Rabbit anti-ERα | Abcam, ab32063 | IF | 1:500 |
| Mouse anti- ERα | Santa, sc-542 | IF | 1:500 |
| Rabbit anti-CD44 | Proteintech, 15675-1-AP | WB | 1:1000 |
|  |  | IF | 1:500 |
| Mouse anti-CD44 | Abcam, 254530 | IF | 1:500 |
| Mouse anti-VIM | Proteintech, 60330-1 | IF | 1:500 |

Abbreviation: WB: Western Blot; IF: Immunofluorescence.

**Table S3.** Primers used for plasmid construction.

| Plasmid | Forward Primer (5’-3’) | Reverse Primer (5’-3’) |
| --- | --- | --- |
| pcDNA3.1(+)-CYP1B1 | CGCGGATCCGAAACCGCACCTCCCCGCA | CCGTCTAGACTTGCTTCTTATTGGCAAGTTTC |
| CRISPRi_CYP1B1 | CACCGAGCGTTGAGATTGAGACTGG | AAACCCAGTCTCAATCTCAACGCTC |
| CRISPRi_lacZ | CACCGCCCGAATCTCTATCGTGCGG | AAACCCGCACGATAGAGATTCGGGC |
