# Supplementary material for: CYP1B1-catalyzed 4-OHE2 promotes the castration resistance of prostate cancer stem cells by estrogen receptor α-mediated IL6 activation
Source: Cell Commun Signal. 2022 Mar 15;20:31. doi: 10.1186/s12964-021-00807-x (PMC8922936; doi:10.1186/s12964-021-00807-x)
Supplement: Supplementary file 3 — Additional file 2. Additional results figures. Figures S1-S12 show additional data related to the results shown in the main figures. Figure S1. The plasmid construction process and plasmid maps. A, pcDNA3.1( +)-CYP1B1 plasmid construction diagram. B-D, Plasmid maps of pcDNA3.1( +)-CYP1B1, CRISPRi-CYP1B1 and CRISPRi-Lac. Figure S2. Transfection efficiency/cytotoxicity of the plasmids. pcDNA3.1( +) plasmids, which expresses 3 × flag-tagged peptide, were transfected into LNCaP cells. The transfection efficiency (A) and cytotoxicity (B) of the plasmids were assessed. Scale Bar = 50 μm. Figure S3. Selected ion monitoring (SIM) chromatograms of the 4-OHE2 standard. E2 was dissolved in methanol. The monitored ion in the mass spectrum had a mass/charge ratio of 755 m/z and a retention time of 6.95 min. Figure S4. Validation of CYP1B1 silencing efficiency in LNCaP-abl and PC3 cells and CYP1B1 overexpression efficiency in LNCaP cells by western blotting. Figure S5. HPLC–MS assay results showing the concentration of intracellular 4-OHE2 in CYP1B1 knockdown or control PC3 cells (t-test). *p < 0.05. Figure S6. The effect of CYP1B1 on the expression of stemness-associated genes. A, Characteristics of differential gene expression profiles of CYP1B1High and CYP1B1Low PRAD samples from the TCGA. B, The expression of some basal cell markers and stemness-associated genes was verified by qRT-PCR in CYP1B1-overexpressing LNCaP cells compared with control LNCaP cells (t-test). C, Representative IF staining of CD44 (red) and CYP1B1 (green) in CRPC tissue. Scale bar = 50 μm. D, The expression of CYP1B1 and stemness-associated genes was tested in spheroid cells (PCSCs) and adherent cells (Bulk) by qRT-PCR (t-test). E–F, Relative mRNA levels of some stemness-associated genes were verified by qRT-PCR in PC3 (E) and LNCaP-abl (F) cells after CYP1B1 knockdown (t-test). All values represent the means ± SD from three independent experiments. *p < 0.05. Figure S7. IL6 expression was tested in sphe [file 12964_2021_807_MOESM3_ESM.docx]

**Additional file 2.**

**Figure S1**

**
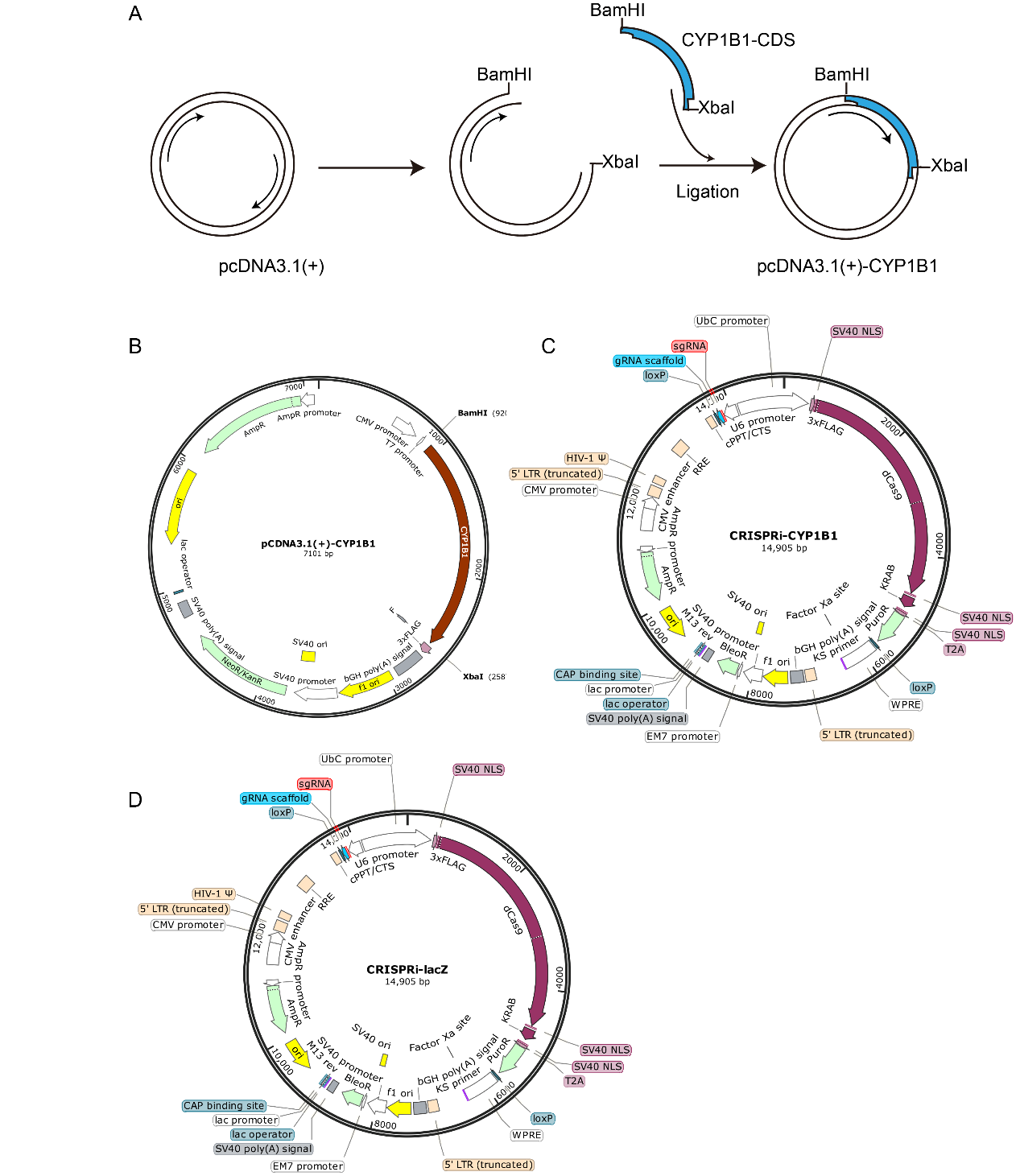
**

**Figure S2**

**
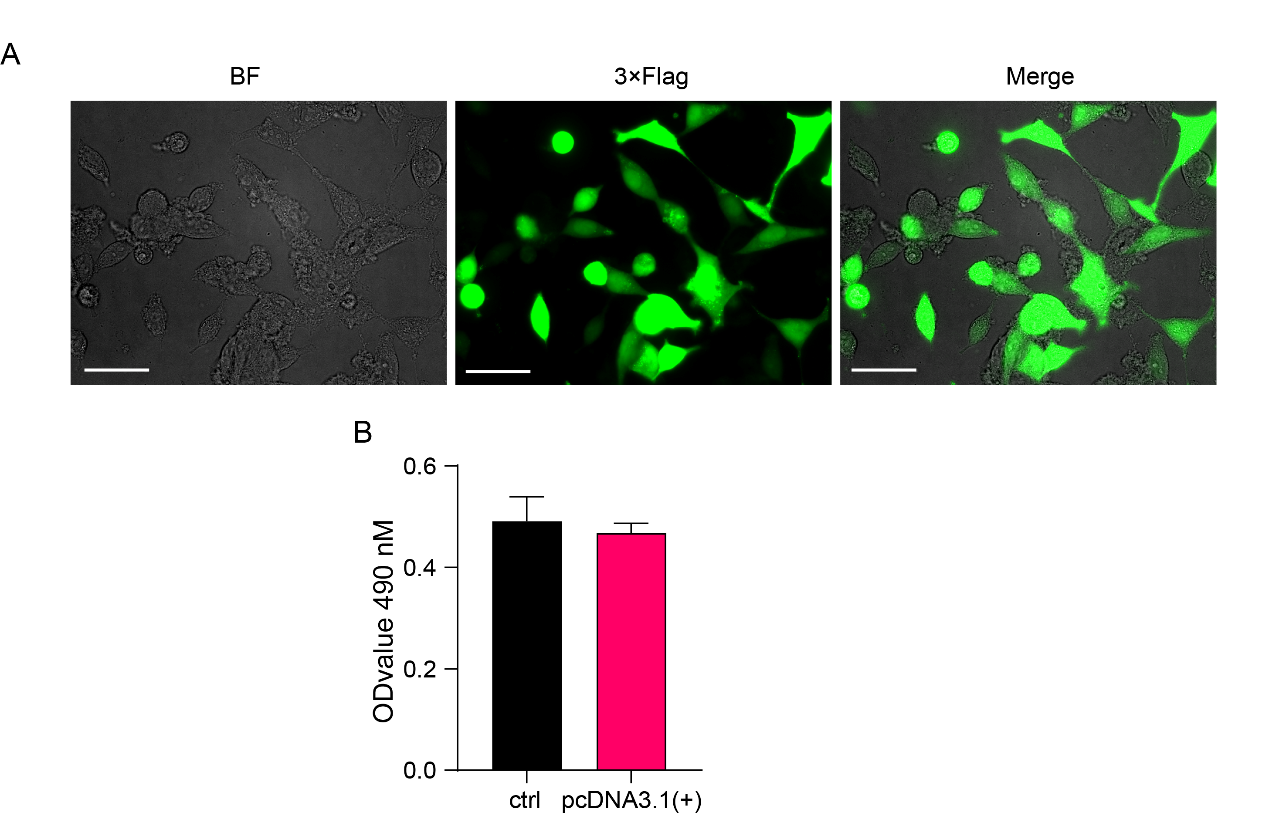
**

**Figure S3**

**
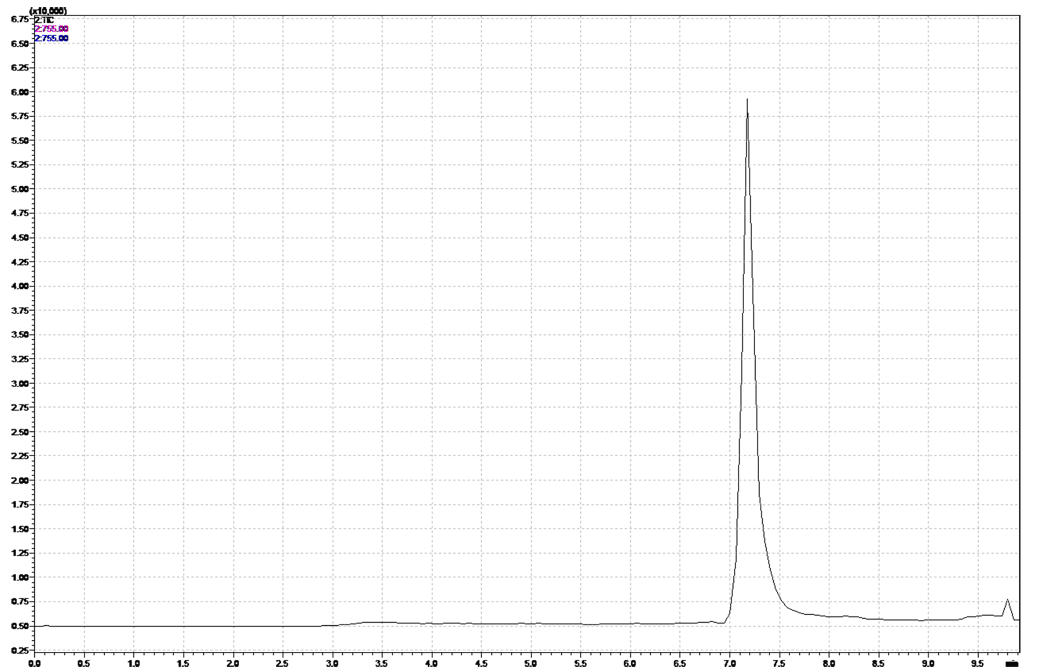
**

**Figure S4**

**
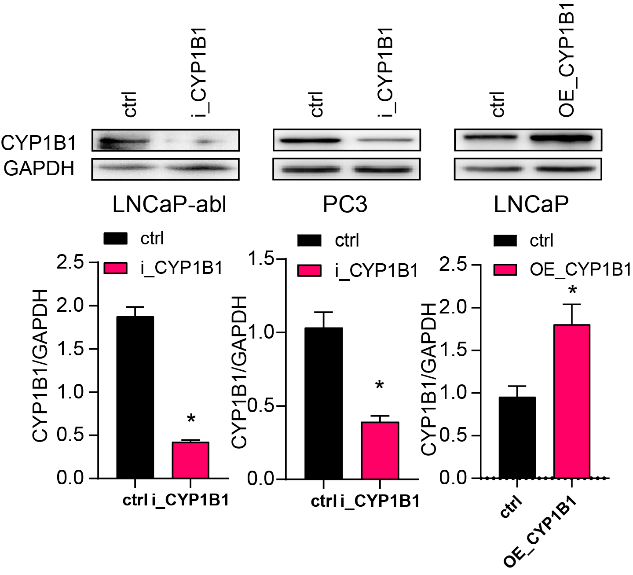
**

**Figure S5**

**
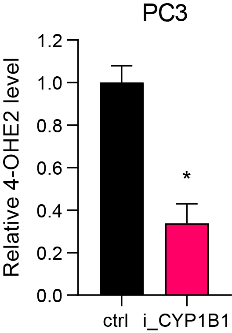
**

**Figure S6**

**
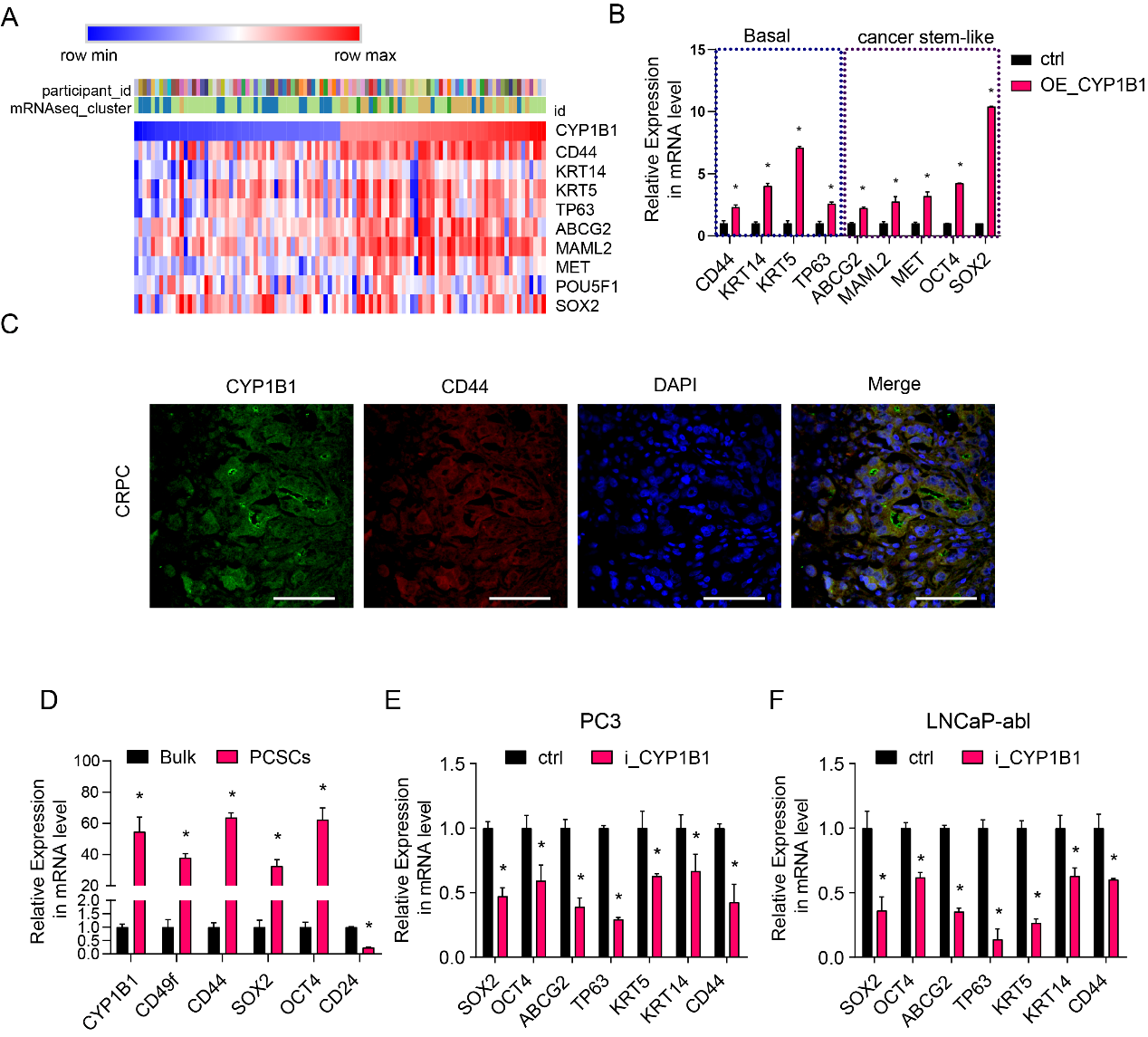
**

**Figure S7**

**
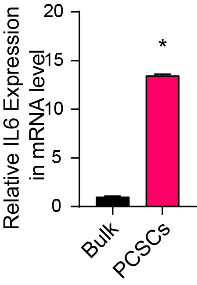
**

**Figure S8**

**
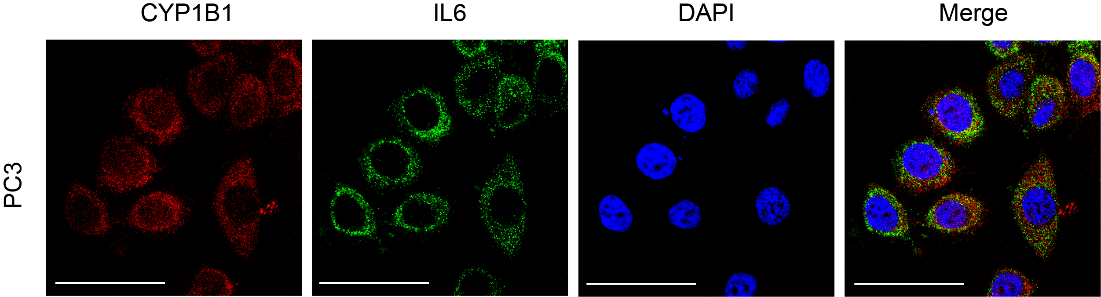
**

**Figure S9**

**
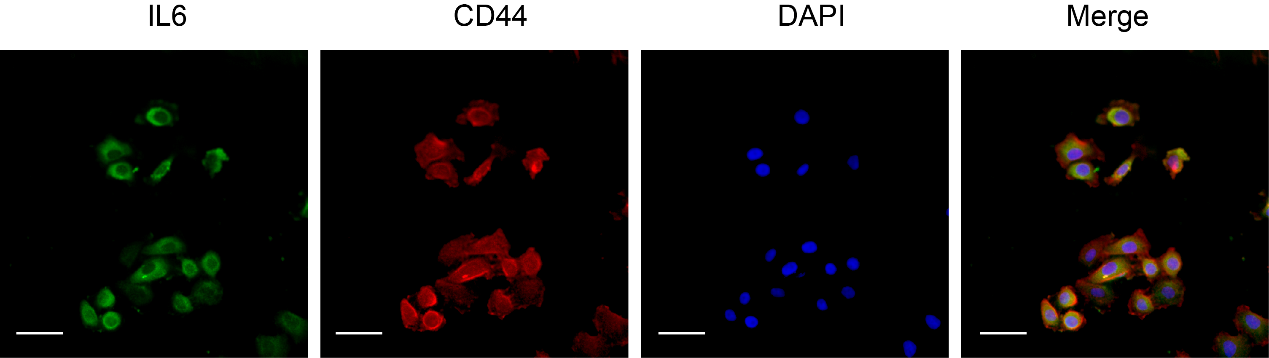
**

**Figure S10**

**
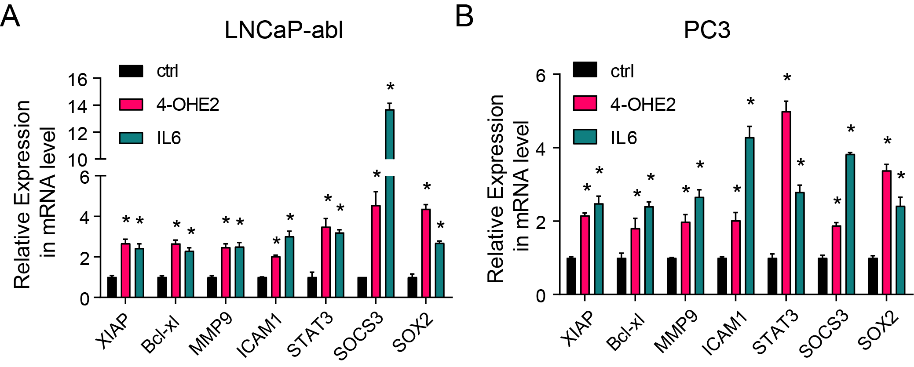
**

**Figure S11**

**
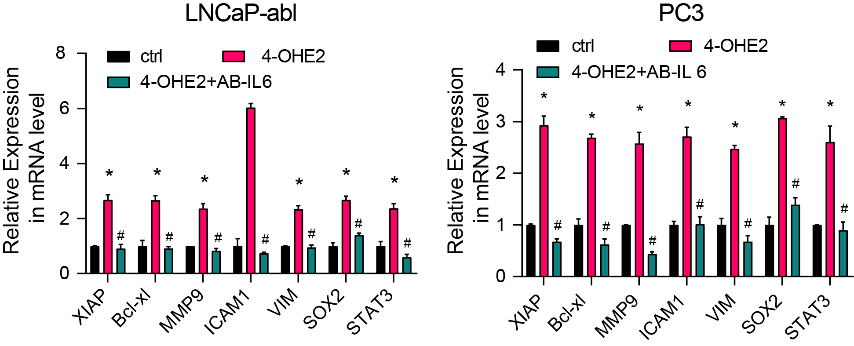
**

**Figure S12**

**
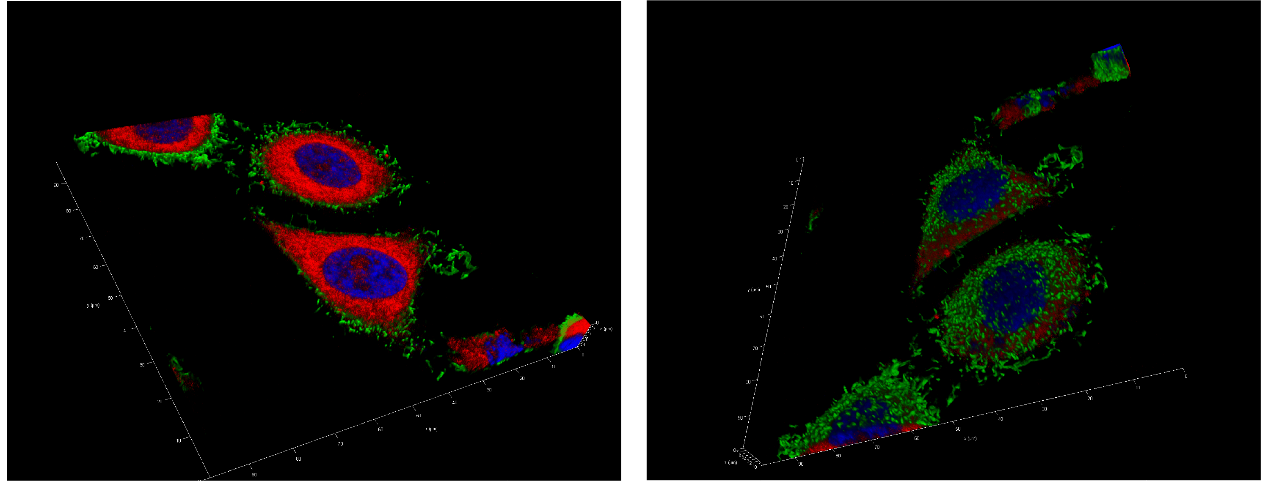
**
